# An ex-vivo quantitative assessment to determine the optimal aortotomy closure technique

**DOI:** 10.1186/s13019-015-0325-7

**Published:** 2015-09-09

**Authors:** Daniel D. Holloway, Jehangir J. Appoo

**Affiliations:** Libin Cardiovascular Institiute, University of Calgary, Room C880, 1403 29th Street NW, Calgary, AB T2N-2T9 Canada

**Keywords:** Cardiovascular surgery, Animal model, Aortic surgery, Surgical technique, Aortotomy

## Abstract

**Background:**

After performing an aortotomy, there are a variety of techniques utilized for suture closure. There is no published data comparing the efficacy of various suture techniques. The goal of this study is to provide an ex-vivo quantitative assessment of resistance to leakage and dehiscence for three aortotomy closure techniques.

**Materials and methods:**

An ex-vivo model was developed utilizing explanted porcine aorta. Aortotomies were closed using one of three techniques: 1) single layer baseball stitch 2) double layer baseball stitch 3) horizontal mattress stitch with a top layer baseball stitch. The aorta was pressurized with saline using an apparatus which captured all leaked fluid. The intra-aortic pressure was adjusted over 8 increments from 110 to 375 mmHg. Leakage rates were determined at each pressure level. Ten aortotomies were performed for each technique, resulting in 240 calculated leakage rates.

**Results:**

At all pressures, the horizontal mattress group was measured to have significantly less leakage when compared to single or double layer baseball stitch closures (*p* < 0.005). There was a trend towards a lower leakage rate in the double layer baseball compared to the single layer baseball stitch. However, this difference is statistically significant only at 300 and 335 mmHg. There were no instances of rupture.

**Conclusion:**

This study provides the first quantitative comparison of three commonly used aortotomy closure techniques. The running horizontal mattress stitch combined with a baseball stitch provides the greatest resistance to leakage at all pressures. This technique may be superior in clinical scenarios with challenging hemostasis.

## Background

Following a surgical aortotomy, there are a wide variety of suture techniques commonly employed for closure. Most commonly, one of three approaches is used: 1) single layer baseball stitch (aka continuous over-and-over stitch); 2) double layer baseball stitch; or 3) horizontal mattress with second layer baseball stitch [1–3]. The specific closure technique used by each surgeon is determined by the surgeon’s preference within the clinical scenario.

Although all of these techniques are commonly used successfully in clinical practice, there is a lack of objective evidence to determine if one technique is superior. Currently, there are no published studies comparing aortotomy closure techniques or recommendations for which approach to take in a given clinical scenario. Each surgeon’s preference appears to be based on their training and personal experiences. Even though they all work well enough to be used clinically, this does not necessarily mean they are all equal in performance. Is there one technique which is most suited for a clinical scenario with very challenging hemostasis such as profound coagulopathy, or an elderly aorta with considerable calcification or stiffness? Furthermore, is there a technique which is most resistant to rupture and would be best suited for scenarios where extreme hypertension is a concern?

The objective of this study was to assess the efficacy of three commonly used aortotomy closure suture techniques under a variety of pressure loading scenarios, so as to inform clinical practice.

## Methods

An ex-vivo model of aortotomy closure was developed using explanted fresh porcine aorta. Descending thoracic aorta was obtained from a local abattoir. All tissues were from young healthy animals slaughtered for consumption. Tissue procurement and use was carried out in strict accordance with the protocol approved by the University of Calgary Animal Care Committee. The descending thoracic aorta was utilized due to its favorable straight length. The aorta was mounted in a purpose-built apparatus to pressurize it with saline.

**Fig. 1:**
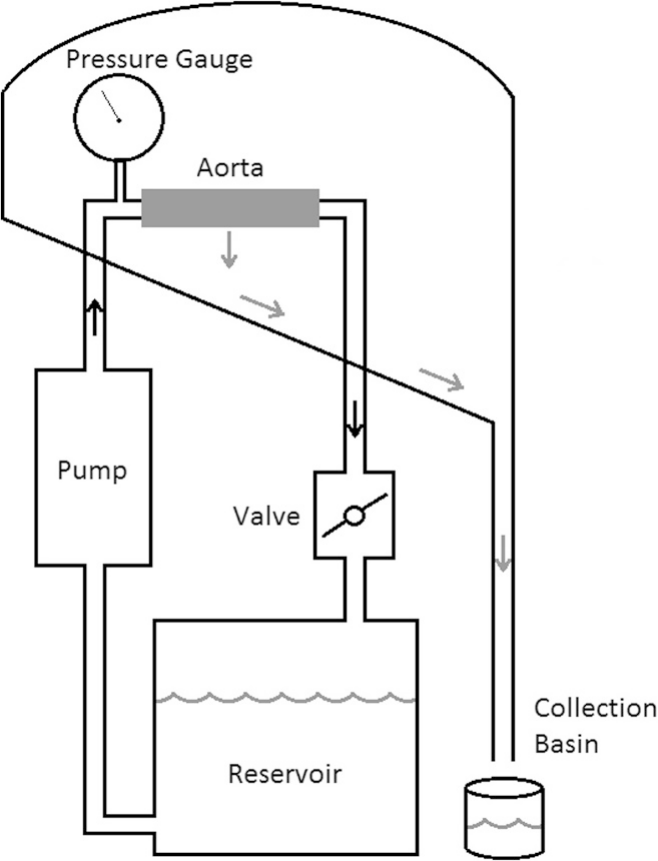
A schematic drawing of the aorta pressurizing apparatus

Depicted in Fig. 1; the apparatus consisted of a reservoir of saline, an electric continuous pump, tubing to and from the segment of mounted aorta, a gauge to measure the pressure at the level of the aorta, and a valve distal to the aorta to regulate outflow and therefore pressure. With the aorta mounted in the apparatus, a continuous closed circuit was created. Fluid which leaked from the aorta was captured by the apparatus and directed to a basin for measurement.

All experiments were conducted by a single surgeon. After mounting an approximately 7 cm long segment of aorta in the apparatus, any intercostal artery branches on the aorta were suture ligated. The baseline leakage rate was calculated by measuring the volume of leaked saline over a period of 5 min at a constant pressure. For each segment of aorta, the baseline leakage rate was determined at 8 pressures: 110, 150, 185, 225, 260, 300, 335, and 375 mmHg. The aortotomy was then performed by conducting a full thickness incision of 2/3 of the aorta. The aortotomy was then closed using one of three techniques: single layer baseball, double layer baseball, horizontal mattress stitch with a top layer baseball stitch. Non-pledgeted 4–0 prolene suture was used for every closure. Following aortotomy closure using one of the three techniques, the leakage rate was again determined at the 8 intra-aortic pressures. The leakage rate due to the aortotomy closure was calculated as the difference between the baseline and post-closure rates. Aorta segments were used for one aortotomy only with each trial receiving a new segment. If a closed aortotomy was believed to have a significant technical error (i.e. a misplaced suture) creating leakage greater than 1000 mL/5 min at any pressure, then that aorta was discarded and replaced. Secondary repair sutures were not utilized. Using this exclusion criteria, two aortas were discarded and replaced.

For each of the three suture techniques 10 segments of aorta were used, each with baseline and post-closure measurements at 8 pressures. The result is a total of 240 aortotomy leakage rates being included for analysis.

Statistical analysis was performed using SPSS (Version 20; IBM, Chicago, IL). If variance was homogenous, as determined by a Levene test with *p* value greater than or equal to 0.05, then comparison of means was performed using ANOVA. If a significant difference was found, then Tukey HSD post-hoc analysis was utilized. If variance was unequal (Levene test *p* value less than 0.05), then comparison of means was performed using Welch-ANOVA. If a significant difference was found, then Games-Howell post-hoc analysis was utilized. A two-sided *p* value less than 0.05 indicated significance.

## Results

The average time required to perform the aortotomy closures was 8.3, 11.4, and 12.8 min for the single layer baseball, double layer baseball, and horizontal with baseball stitch, respectively. There was a trend of increasing duration from the single baseball, to the double baseball, to the horizontal with baseball stich. However, the difference falls short of statistical significance (*p* = 0.074).

**Fig. 2:**
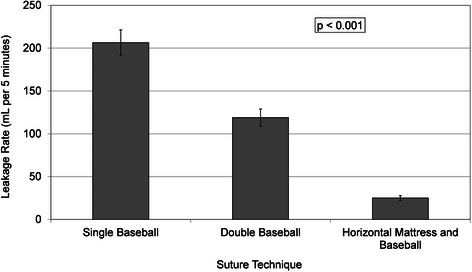
Mean leakage rate for all pressures combined for each of the three aortotomy closure techniques. Error bars indicate standard error

The mean leakage rate for all pressures combined for each suture technique are presented in Fig. 2. There is a highly significant difference in mean leakage rates (*p* <0.001). Post-hoc analysis found a significant difference between all three groups with *p* <0.001 for all two-way comparisons. The double layer horizontal mattress with running baseball stitch provided the lowest leakage rate, followed by the double layer baseball stitch. The single layer baseball stitch had the highest leakage rate.

**Fig. 3:**
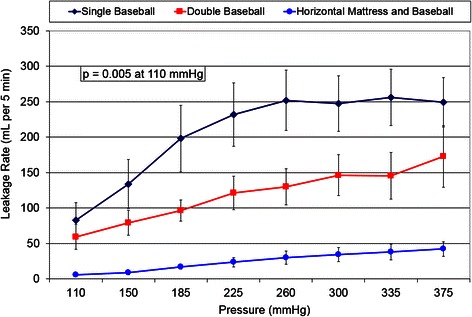
Leakage rates for each closure technique at each pressure. Error bars indicate standard error

The leakage rates for each suturing technique at each pressure are presented in Fig. 3. The associated *p* values are presented in Table 1. At each pressure, there was a significant difference between the groups. Post-hoc analysis found that at all pressures, the horizontal mattress with baseball stitch produced significantly lower leakage rates than both the single layer baseball and double layer baseball closures. Comparison of the single and double baseball techniques found that there was a trend towards a lower leakage rate in the double layer closure. However, it was statistically significant only at pressures of 300 and 335 mmHg.

**Table 1:** Results of comparison of means and post-hoc analysis for each pressure

Pressure (mmHg)	Levene	ANOVA	Welch-ANOVA	Single to double	Single to horizontal	Double to horizontal
110	0.001		0.005	0.730	0.031	0.037
150	0.002		0.001	0.366	0.014	0.007
185	0.002		<0.001	0.143	0.010	0.001
225	0.003		<0.001	0.111	0.003	0.006
260	0.009		<0.001	0.065	0.001	0.009
300	0.045	<0.001		0.047	<0.001	0.026
335	0.086	<0.001		0.041	<0.001	0.049
375	0.032		<0.001	0.374	<0.001	0.037
Combined	<0.001		<0.001	<0.001	<0.001	<0.001

Throughout this study, there were no instances of frank rupture or suture dehiscence resulting in sudden increases in leakage.

## Discussion

In this study, a two layer aortotomy closure using a horizontal mattress stitch followed by an over-and-over baseball stitch was superior to both single and double layer baseball stitches with respect to resistance to leakage. This superiority was evident at all measured pressures. The results support the anecdotal findings of Tsuji et al. who claimed it to have superior hemostasis compared to single baseball, double baseball, interrupted figure-of-eight, and interrupted mattress sutures [3].

The reason for the decreased leakage was not directly investigated in this study. However, the previously proposed benefit of the horizontal mattress stitch distributing the tension across the tissue and thereby decreasing bleeding from needle holes remains the most likely explanation [3]. Furthermore, unlike the other techniques where all the needle holes are exposed to blood at the aortic intra-luminal pressure, the top layer in this technique is isolated and therefore reinforces tissue approximation without additional needle holes into the aortic lumen. We believe this isolated suture line to be a major contributor to the superiority of this technique.

A proposed advantage of a single layer technique is that it can be performed in less time. This study does indeed show a trend where the single layer took the least amount of time. However, the time difference was not statistically significant. More importantly though is whether the shorter duration is clinically significant. A double layer closure added on average about 4 min to what would be the cross-clamp time.

This study has compared three techniques that are routinely being used to close aortas. These techniques all work well enough to be used clinically but this does not mean they are equal. The two layer horizontal with baseball stitch was clearly superior on this static ex-vivo model. The question remains as to whether this superiority translates to the operating room with a benefit for the patient. In clinical scenarios such as profound coagulopathy, thin diseased aorta, or atherosclerotic non-compliant fragile aortas where hemostasis can be challenging, it may be advantageous to use the technique with the most inherent resistance to leakage. With the absence of platelets, absence of clot, and using saline with a lower viscosity than blood, this ex-vivo model subjected the aorta to a challenging environment for hemostasis. In this environment the horizontal mattress with baseball closure technique exhibited the most inherent resistance to leakage. Aside from a slightly longer duration to perform it, there does not appear to be a significant disadvantage of this technique. For these reasons it appears to be the best choice of technique for challenging conditions and also a reasonable choice for routine aortotomy closure.

In regards to rupture or dehiscence, we did not see any. There was therefore no observed difference in the three techniques, even at supra-physiologic pressures. However, it is important to note that this is in the context of static pressures. This finding is similar to the aortic dissection investigations reported in 1970 by Prokop et al. [4] where non-pulsatile pressure did not propagate dissection in either synthetic or canine aorta; even at pressures of 400 mmHg. In contrast, pulsatile flow with a pulse wave with a high pressure change (dp/dt_max_) was associated with dissection propagation [4]. More recent evidence shows that the amplitude of the pressure wave as well as the number of cycles of exposure contributes to propagation of aortic dissection [5]. Although the data in the literature is related to aortic dissection, it is plausible that the same mechanism could result in suture dehiscence at the aortotomy site. In order to further test the suture techniques for a difference in resistance to rupture or dehiscence, a dynamic model with systolic and diastolic pressures will need to be utilized.

### Limitations

The lack of pulsatile pressure limits the conclusions related to resistance of rupture and dehiscence. Future studies with a pulsatile model will need to be performed. Furthermore, we did not attempt to compare the mechanical properties of the closed aortotomies. The tensile strength of the suture-lines and porcine tissue were not measured. However, as we did not experience any rupture or dehiscence, it is evident that the strength exceeded the demand from this static model.

The use of saline instead of blood creates a model with a propensity to leak, more so than seen clinically. The disadvantage of this is that characteristics which lead to leakage in a saline model may be minimized clinically with the hemostatic properties of blood and topical agents. However, the advantage of a leaky model is that it enables a comparison of each technique’s inherent resistance to leakage in a worst-case scenario. Another limitation of this model is that it used dead, explanted tissue from otherwise healthy young pigs. The aorta did not have atheroma or calcification as commonly seen in human patients. The aortas appeared grossly normal in form and function in all cases although histologic examinations were not performed. However, to our knowledge this is the only quantitative comparison of aortotomy closure techniques and it represents a starting point for further investigation.

## Conclusions

Three commonly used aortotomy closure techniques were compared using an ex-vivo porcine model. The two layer technique utilizing a horizontal mattress stitch followed by a baseball stitch produced the lowest leakage rates at all pressures. This technique may be favorable for clinical scenarios where hemostasis is a particular concern. It is also a good choice for all routine aortotomy closures as the time increase over a single layer closure is likely clinically insignificant. All three closure techniques proved equally durable at resisting rupture or dehiscence at all measured static pressures although a pulsatile model will be required to test the effect of a pressure wave.
